# Secondary Malignancy Risk Following Proton vs. X-ray Treatment of Mediastinal Malignant Lymphoma: A Comparative Modeling Study of Thoracic Organ-Specific Cancer Risk

**DOI:** 10.3389/fonc.2020.00989

**Published:** 2020-07-07

**Authors:** Laila König, Peter Haering, Clemens Lang, Mona Splinter, Bastian von Nettelbladt, Fabian Weykamp, Philipp Hoegen, Jonathan W. Lischalk, Klaus Herfarth, Jürgen Debus, Juliane Hörner-Rieber

**Affiliations:** ^1^Department of Radiation Oncology, Heidelberg University Hospital, Heidelberg, Germany; ^2^Heidelberg Institute of Radiation Oncology (HIRO), Heidelberg, Germany; ^3^National Center for Tumor Diseases (NCT), Heidelberg, Germany; ^4^Department of Radiation Oncology, Heidelberg Ion-Beam Therapy Center (HIT), Heidelberg University Hospital, Heidelberg, Germany; ^5^Clinical Cooperation Unit Radiation Oncology, German Cancer Research Center (DKFZ), Heidelberg, Germany; ^6^Department for Medical Physics in Radiation Oncology, German Cancer Research Center (DKFZ), Heidelberg, Germany; ^7^German Cancer Consortium (DKTK), Heidelberg, Germany; ^8^Department of Radiation Medicine, Georgetown University School of Medicine, Washington, DC, United States

**Keywords:** mediastinal lymphoma, proton radiotherapy, intensity modulated radiotherapy, photon radiotherapy, secondary malignancies, risk

## Abstract

**Purpose:** Proton radiotherapy (PRT) is potentially associated with a lower risk for secondary malignancies due to a decreased integral dose to the surrounding organs at risk (OARs). Prospective trials confirming this are lacking due to the need for long-term follow-up and the ethical complexities of randomizing patients between modalities. The objective of the current study is to calculate the risk for secondary malignancies following PRT and photon-based intensity-modulated radiotherapy (IMRT).

**Materials and Methods:** Twenty-three patients (16 female and seven male), previously treated with active scanning PRT for malignant mediastinal lymphoma at Heidelberg Ion Beam Therapy Center, were retrospectively re-planned using helical photon IMRT. The risk for radiation-induced secondary malignancies was estimated and evaluated using two distinct prediction models (1–4).

**Results:** According to the Dasu model, the median absolute total risk for tumor induction following IMRT was 4.4% (range, 3.3–5.8%), 9.9% (range, 2.0–27.6%), and 1.0% (range, 0.5–1.5%) for lung, breast, and esophageal cancer, respectively. For PRT, it was significantly lower for the aforementioned organs at 1.6% (range, 0.7–2.1%), 4.5% (range, 0.0–15.5), and 0.8% (range, 0.0–1.6%), respectively (*p* ≤ 0.01). The mortality risk from secondary malignancies was also significantly reduced for PRT relative to IMRT at 1.1 vs. 3.1% (*p* ≤ 0.001), 0.9 vs. 1.9% (*p* ≤ 0.001), and 0.7 vs. 1.0% (*p* ≤ 0.001) for lung, breast, and esophageal tumors, respectively. Using the Schneider model, a significant risk reduction of 54.4% (range, 32.2–84.0%), 56.4% (range, 16.0–99.4%), and 24.4% (range, 0.0–99.0%) was seen for secondary lung, breast, and esophageal malignancies, favoring PRT vs. X-ray-based IMRT (*p* ≤ 0.01).

**Conclusion:** Based on the two prediction models, PRT for malignant mediastinal lymphoma is expected to reduce the risk for radiation-induced secondary malignancies compared with the X-ray-based IMRT. The young age and the long natural history of patients diagnosed with mediastinal lymphoma predisposes them to a high risk of secondary malignancies following curative radiotherapy treatment and, as a consequence, potentially reducing this risk by utilizing advanced radiation therapy techniques such as PRT should be considered.

## Introduction

Over the last few decades, significant improvements in combined modality therapy consisting of multi-agent chemotherapy and consolidation radiotherapy (RT) have resulted in high cure rates in patients diagnosed with lymphoma. Furthermore, due to their young age and excellent survival rates, the mediastinal lymphoma patients are at a significant risk for late toxicity from their oncologic therapy. Notable improvements in oncologic outcomes have prompted a new focus on the reduction of treatment-related morbidity *via* de-escalation in both the chemotherapy and the radiation realms. A reduction in RT treatment doses and field sizes, as well as the utilization of modern highly conformal RT techniques [e.g., intensity-modulated RT (IMRT), in contrast to conventional 3D-conformal radiotherapy], has led to a further reduction in radiation doses to organs at risk (OARs) (5–8). Thoracic radiotherapy to the mediastinum poses notable challenges due to the close proximity of target volumes to OARs including the heart, breast, and esophagus, making dose reductions to these organs difficult despite using the most advanced X-ray-based radiotherapy techniques such as IMRT. Multiple comparative dosimetric studies have demonstrated radiation dose reductions to healthy surrounding tissues due to the superior physics of proton therapy *vis*-à-*vis* the Bragg Peak (9–12). Radiobiologically, these dose reductions can not only result in reduced deterministic side effects leading to lower acute toxicity rates but also in reduced stochastic side effects and, consequently, reduced risk for secondary malignancies (SM). Due to the stochastic nature of the risks, even small doses delivered to OARs may induce a long-term SM induction after RT. However, prospective trials confirming this are lacking due to the need for an extremely long-term follow-up and the ethical complexities of randomizing patients between these two modalities. Although the risk for development of SM is small, it is statistically significant, particularly for long-term survivors of treatment, e.g., lymphoma patients (7, 8). One study conducted with extended follow-up, published by Sethi et al., reported statistically significant reductions in secondary malignancy risk in pediatric patients treated for retinoblastoma (0 vs. 14%, *p* = 0.015) (13). The frequency of radiation-induced cancers after total body exposures with very low doses of ionizing radiation has been determined in different epidemiological studies (14, 15). However, these epidemiologic data involve doses (<100 mSv) which are dramatically lower than those used for RT. Hence, different dose–response models, valid for all dose levels, have been proposed using mechanistic models for predicting cancer induction after fractionated radiotherapy, which are based upon the linear–quadratic model:

(1) The Dasu model (1) explores several methods for estimating the risk of cancer following RT in order to investigate the influences of fractionation and non-uniformity of dose to the irradiated volume. This model takes into consideration the competition between cell killing and the induction of carcinogenic mutations for a more realistic risk estimate.
(2) The Schneider model introduced the concept of organ equivalent dose (OED) to estimate organ-specific radiation-induced cancer incidence rates (4). The OED concept assumes that any two dose distributions in an organ are equivalent if they cause the same radiation-induced cancer incidence. The two operational parameters of the OED concept are the organ-specific cancer incidence rate at low doses, which was taken from the data of atomic bomb survivors, and cell sterilization at higher doses. For the OED concept, the effect of cell sterilization in various organs was estimated by analyzing the historical secondary cancer incidence data of patients treated with RT due to Hodgkin's disease. Using these two model parameters, the OED concept can be applied to any three-dimensional dose distribution for estimating radiation-induced secondary malignancy incidence.

The aim of the present study was to use these two radiobiological models to investigate the potential improvement of PT vs. X-ray irradiation relative to the risk of radiation-related secondary malignancies using actual proton dosimetric data from patients who were previously treated with mediastinal RT for malignant lymphoma.

## Materials and Methods

### Patient Selection and Treatment Planning

Twenty-three (16 female and seven male) patients with histologically proven lymphoma with mediastinal involvement and treated with consolidative proton radiotherapy were included in the present study. The patients received PT due to their young age (<30 years), in female patients with an expected high dose to breast tissue (*D*_mean_ > 4.5 Gy) and/or in patients with particularly high expected radiation dose to the heart (*D*_mean_ > 5 Gy) if treated with conventional photon irradiation. In summary, 10 patients with bulky disease (>7.5 cm) non-Hodgkin lymphoma (NHL) received consolidation RT following induction chemotherapy consisting of R-CHOP+/−MTX (16– 18). Thirteen Hodgkin lymphoma (HL) patients were treated according to the German Hodgkin Study Group criteria, depending on the stage and the risk factors (2, 19, 20). Treatment technique and clinical outcomes have recently been described in detail (12). The patient, treatment, and disease-specific characteristics are presented in Table 1.

**Table 1:** Patient, treatment, and disease-specific characteristics of 23 patients with mediastinal lymphoma.

Number of patients	23
Median age (range)	30 years (18-54 years)
Sex (m/f)	7/16
HL/NHL	13/10
Ann Arbor staging	
I	3 (13%)
II	13 (57%)
III	0 (0%)
IV	7 (30%)
Median total dose (range)	36 Gy(RBE) [20-39.6 Gy(RBE)]
Median no. of fractions (range)	18 (10-22)
Median dose per fraction (range)	2 Gy(RBE) [1.8-2 Gy(RBE)]
Median PTV	494 ml (120-886 ml)
Mediastinal involvement	
Only superior	10 (43%)
Superior and inferior	13 (57%)
Laterality	
Left	8 (35%)
Right	9 (39%)
Middle	6 (26%)
Additional cervical involvement	8 (35%)

*Gy(RBE), Gray (Relative Biological Effectiveness); PTV, Planning target volume*.

For treatment planning, the patients were immobilized with the help of either individually shaped thermoplastic masks with shoulder fixation or the WingSTEP system (IT V, Innsbruck). A planning computed tomography (CT) scan with 3-mm slice thickness as well as a 4D CT scan under free breathing were acquired using Siemens' either Somtom or Confidence (Siemens Healthnears, Erlangen Germany). The aim of the 4D CT was to qualitatively analyze the impact of respiratory motion on tumor movement. Particle therapy planning was performed using Siemens Syngo PT Software (Siemens, Erlangen, Germany) that applies pencil beam algorithm for dose calculation (21–23). The prescribed dose was optimized with proton beams of spot size of 8–25 mm full width at half maximum, and with 2–3 mm of overlap in lateral (dx, dy) and longitudinal (dz) directions. Both single-beam optimization and multi-beam optimization (IMPT) were applied, depending on the different tumor locations. If IMPT was applied, generation of high-dose gradients per field was avoided. Due to the location of the clinical tumor volumes (CTV)s in close proximity to the lungs, a maximum of two anterior beams with gantry angles between ± 20° was selected. CTV coverage with D95% to 95% of the prescribed dose was aimed while respecting known OAR dose constraints (24). The final proton dose was scaled with a constant radiobiological effectiveness (RBE) factor of 1.1. An active beam application with raster-scanning technique (25) under daily image guidance was used.

Comparative photon plans were calculated for all patients using the TomoTherapy® Treatment Planning System (Tomotherapy, Accuray® Incorporated, Sunnyvale, USA). Whenever possible, directional or complete blocks for breast tissue were used for optimization, resulting in a “butterfly” IMRT beam arrangement approach [weighted anteriorly and posteriorly oblique beam entry angles (26)]. The planning goals were the same for the proton and the IMRT plans, with the aim to keep the dose to the surrounding OAR as low as reasonably achievable and not only according to QUANTEC and Emami constraints, which can be easily achieved in moderate-dose prescriptions like lymphoma treatments. Since this young patient cohort was treated on a solely curative basis, main priority was always given to optimal target coverage. Further prioritization depended on the anatomical localization (upper vs. lower mediastinal region with precardial involvement) and the gender of the patient, but with a generally higher priority to breast and heart tissue compared to the lung and the esophagus.

### Risk Estimation for Radiation-Induced Secondary Cancers

Two distinct radiobiological models proposed by Dasu et al. (1) and Schneider et al. (4) were applied for the risk estimation of radiation-induced secondary cancers as previously described by Mondlane et al. (27). Data extracted from the dose–volume histograms from both the proton and the X-ray plans were used for the risk calculation of radiation-induced secondary malignancies.

### Dasu Model

The Dasu model is a linear–quadratic (LQ)-based model (Equation 1):

$$\begin{array}{l} Total\;risk_{organ}=\frac{1}{\underset{i}{\sum }v_{i}}\underset{i}{\sum }v_{i}\\ \;\times \left\{({\alpha_{1}D}_{i}+\frac{\beta_{1}D_{i}^{2}}{n})\times exp[-(\alpha_{2}D_{i}+\frac{\beta_{2}D_{i}^{2}}{n})]\right\} \end{array}$$

where *v*_*i*_ is the volume of tissue receiving dose *D*_*i*_ given in *n* fractions. The first term in the parenthesis describes the induction of DNA mutations, while the second term models cell survival in the irradiated organs. Calculations of the parameter α_1_ were performed with the risk coefficients for fatal and total risk of cancer induction derived according to the recommendations of ICRP Publication 103 as previously described (1, 27, 28) (see Table 2). The term “total risk” defines the mere risk for development of cancer, while the term “fatal risk” describes the risk of induced secondary cancer leading to death. An α/β ratio of 3 was taken for the lungs, esophagus, and breasts. Nominal risk coefficients are derived by averaging sex and age at exposure lifetime risk estimates in representative populations.

**Table 2:** Risk coefficients (α_1_, second and third column) and the linear quadratic model parameter (last column) used for risk assessment for the different organs at risk.

**Organ**	****α_1_** (Gy ^**−1**^) fatal risk**	****α_1_** (Gy ^**−1**^) total risk**	****α_2_** (Gy ^**−1**^)**
Lung	0.0101	0.0144	0.129
Breast	0.0028	0.0144	0.008
Esophagus	0.0014	0.0015	0.274

*The risk coefficients were taken from ICRP 103, the linear LQ-model parameters were adapted from Schneider et al. (4)*.

### Schneider-Model

The risks for inducing secondary malignancies were also estimated using the Schneider model, which is based on determination of the OED (Equation 2):

$$\begin{array}{l} OED=\frac{1}{\underset{i}{\sum }v_{i}}\underset{i}{\sum }v_{i}\times RED\left(D_{i}\right) \end{array}$$

where *v*_*i*_ and *D*_*i*_ are defined as in the Dasu model and RED (*D*_*i*_) is the selected dose–response relationship.

As described by Mondlane et al. (27), three distinct dose–response relationship scenarios (linear, linear–exponential, and plateau) were applied for estimating the risk of SM. The linear model assumes a direct increase in risk with increasing doses. The linear–exponential dose relationship completely neglects the repopulation/repair effect, while the plateau model expects complete repopulation/repair to take place. The aforementioned three equations modeling the dose–response relationship for linear, linear–exponential, and plateau models are depicted in Equation (3).

$$\begin{array}{l} RED\left(D_{i}\right)=\{\begin{array}{c} D_{i}\\ D_{i}e^{-\alpha^{\prime }D_{i}}\\ \frac{1-e^{-\alpha^{\prime }D_{i}}}{\alpha^{\prime }} \end{array} \end{array}$$

According to Mondlane et al. (27), α′ is defined by applying the LQ model and is proportional to the number of cells which are reduced by cell killing:

$$\begin{array}{l} \alpha^{\prime }=\alpha +\beta \frac{D_{i}}{n} \end{array}$$

in which *n* is the number of fractions used. The values of α in Equation (4) are shown in the last column of Table 2 as α_2_. Analogously to the Dasu model, an α/β ratio of 3 was taken for the lungs, esophagus, and breasts. The relative risks for SMs were calculated as the ratio of the OEDs obtained for specific OARs (the PT dose relative to the photon dose). Therefore, a value <1 stands a lower risk for SM induction following PT.

### Follow-Up

Following the completion of thoracic proton radiotherapy, the patients received regular follow-up visits including clinical examinations and CT or MR imaging. Response to treatment was assessed using the revised response criteria for lymphoma (29).

### Statistical Analysis

Statistical comparisons were performed using the non-parametric Wilcoxon signed-rank test. Significance was noted for two-tailed *p*-values of ≤ 0.05. Survival analyses for overall (OS) as well as progression-free survival (PFS) following radiotherapy were performed using the Kaplan–Meier method. A *p*-value ≤ 0.05 was considered as statistically significant. All statistical analyses were performed using the software SPSS 24.0 (IBM Corporation, Armonk, NY, USA).

### Ethical Approval

Ethical approval was obtained from the local Ethics Committee of Heidelberg University Hospital (S-201/2017).

## Results

### Patient and Treatment Characteristics

Twenty-three patients with a median age of 30 years (range, 18–54 years) and diagnosed with mediastinal lymphoma were treated with consolidation radiotherapy using PT. Fifty-seven percent (*n* = 13) of the patients suffered from HL, whereas 43% (*n* = 10) of the patients had aggressive NHL. Most patients presented in Ann Arbor stages I–II (70%) with involvement of the superior and the inferior mediastinal regions (57%). Additional cervical involvement was present in one third of the patients. Median treatment volume (planning target volume) was 494 ml (range, 120–886 ml). Complete patient-, treatment-, and disease specific characteristics are shown in Table 1.

### Planning and Dosimetric Characteristics

**Figure 1:**
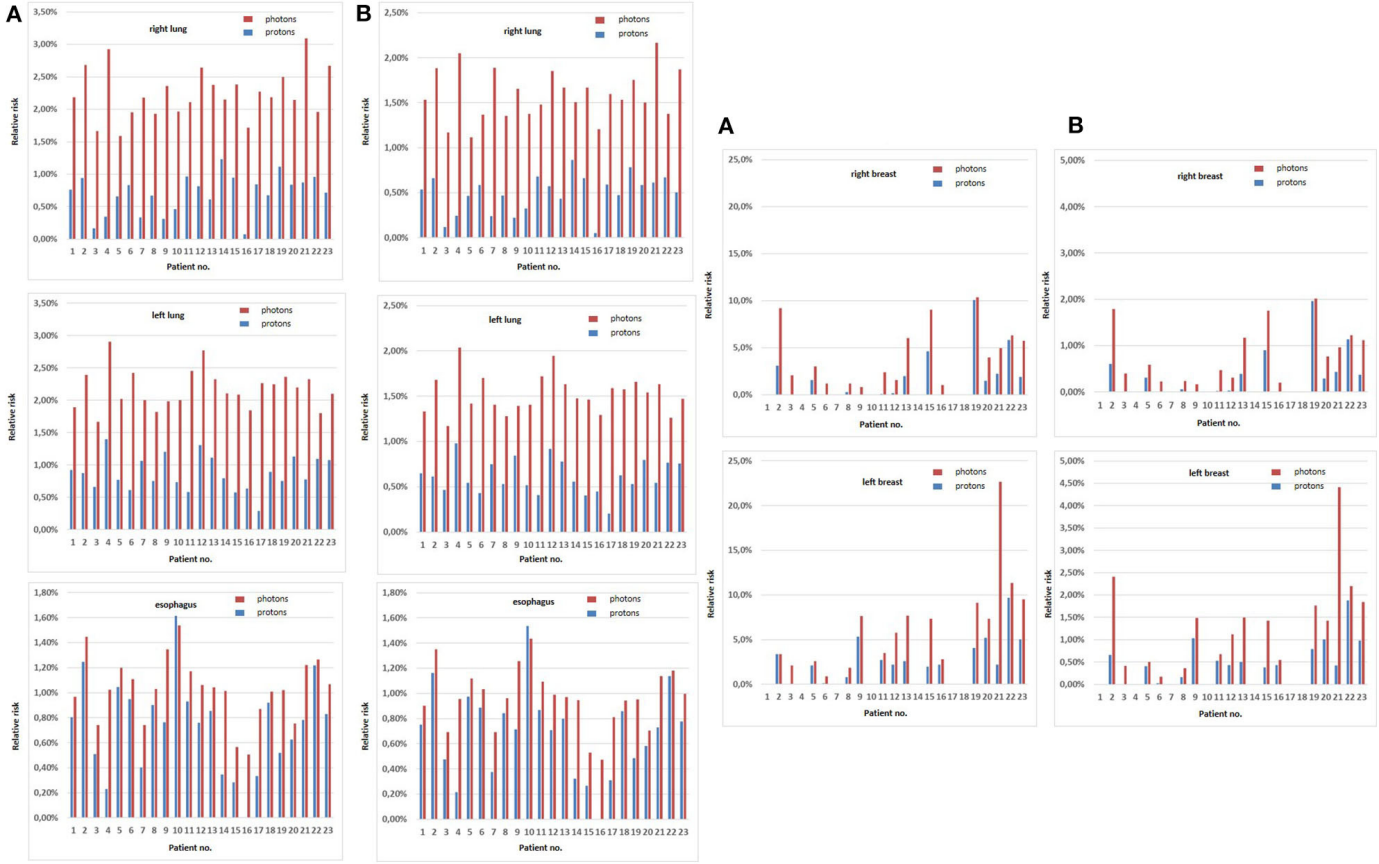
Total **(A)** and fatal **(B)** secondary malignancy risks according to the Dasu model for relevant thoracic organs (right and left lung, esophagus, right and left breast) for each patient. Total and fatal secondary malignancy risks for photons are depicted in red, for protons in blue.

The Dasu model was applied to estimate both the total risk as well as the fatal risk. Figure 1 depicts the calculated risks for total and fatal SM induction for relevant thoracic organs for each patient. For X-ray irradiation, the median total risk for tumor induction was calculated to be 2.2% (range, 1.6–3.1%), 2.1% (range, 1.7–2.9%), and 1.0% (range, 0.5–1.5%) for the right lung, the left lung, and the esophagus, while for proton irradiation the risk was significantly reduced to 0.8% (range, 0.1–1.2%), 0.8% (range, 0.3–1.4%), and 0.8% (range, 0.0–1.6%), respectively (*p* ≤ 0.001). The fatal risk for secondary malignancies also significantly decreased to 0.5% (range, 0.1–0.9%), 0.6% (range, 0.2–1.0%), and 0.7% (range, 0.0–1.5%) in the right lung, the left lung, and the esophagus when applying PT, compared to 1.5% (range, 0.7–2.2%), 1.5% (range, 0.6–2.0%), and 1.0% (range, 0.5–1.4%) with photon irradiation (*p* ≤ 0.001). For female patients treated with PT, the risk of total and fatal cancer induction was 1.5% (range, 0–10.1%) and 0.3% (range, 0–2.0%) for the right breast as well as 2.4% (range, 0–9.7%) and 0.5% (range, 0–1.9%) for the left breast, respectively. A significant increase in both total cancer and fatal cancer induction was calculated in the corresponding photon plans with 3.5% (range, 0.8–10.4%) and 0.7% (range, 0.2–2.0%) for the right breast and 6.6% (range, 0.9–22.7%) and 1.4% (range, 0.2–4.4%) for the left breast, respectively (*p* ≤ 0.001). However, one patient showed a slightly increased risk for both total and fatal esophagus cancer induction when PT was applied compared to photon irradiation (patient 10, Figure 1).

**Figure 2:**
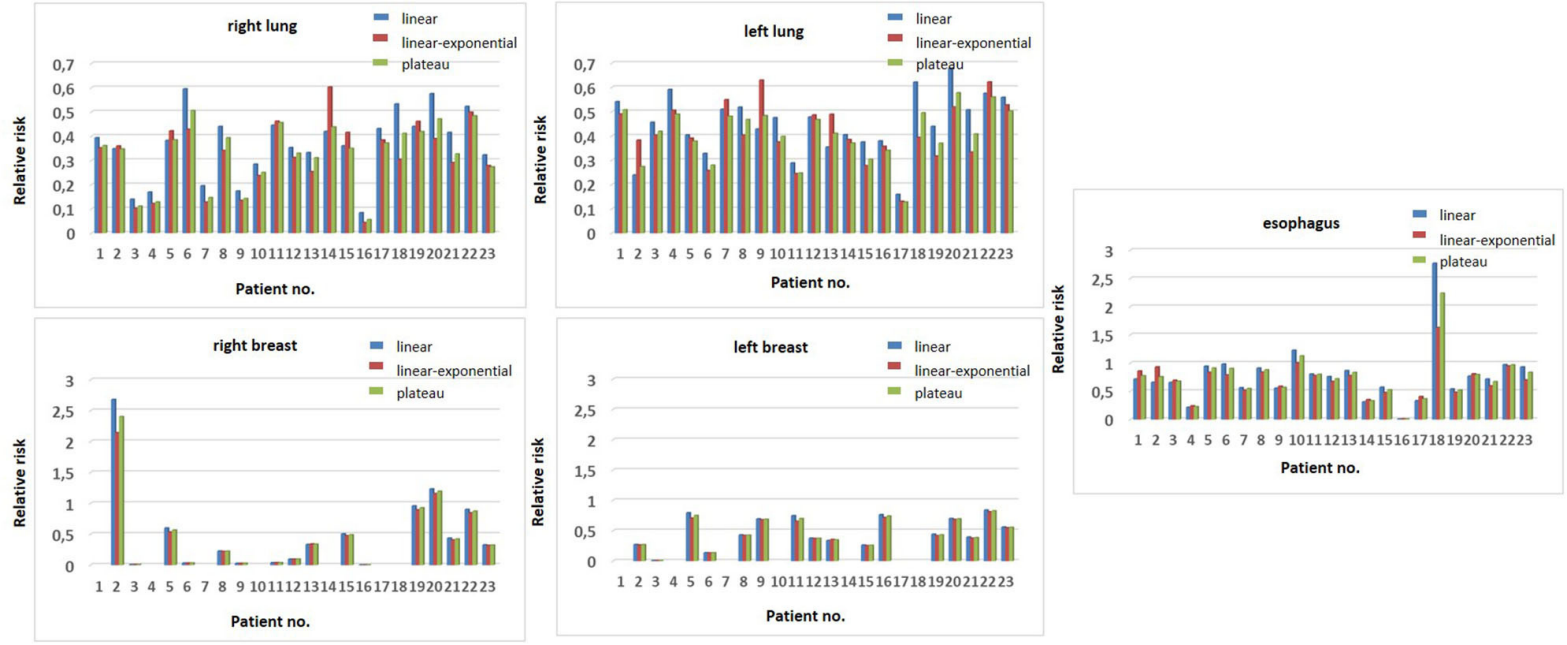
Relative risk reduction for the three distinct dose-response relationship models (linear in blue, the linear-exponential in red and the plateau model in green) according to the Schneider model. Calculated relative risks for tumor induction are shown for relevant thoracic organs (right and left lung, esophagus, right, and left breast) for each patient.

Utilizing the Schneider model to estimate the risk for carcinoma induction, the ratios of the OED values derived from the PT and the X-ray therapy plans were calculated and the relative risk reduction using the linear, the linear–exponential, and the plateau model was derived. According to all three models, PT statistically significantly reduced the risk of radiation-induced lung, esophagus, and breast carcinoma for female patients (at least *p* ≤ 0.008) when compared to X-ray irradiation (Table 3). For each patient, the calculated relative risks for tumor induction in bilateral lungs, esophagus, and bilateral breasts for female patients are presented in Figure 2 for the three distinct dose–response relationship models. However, two patients (patients 10 and 18) were calculated to have an increased relative risk for esophageal cancer and two female patients showed a higher relative risk for right-sided breast cancer (patients 2 and 20) for PT compared to X-ray radiotherapy.

**Table 3:** Median values (range) of the relative risks for observing carcinomas at OAR (lung, breast, esophagus) assessed using the Schneider-model.

	**Proton/Photon relative risk of cancer**					
	**Linear**	***p*-value**	**Exponential**	***p*-value**	**Plateau**	***p*-value**
Lung right	0.38 (0.08-0.60)	<0.001	0.34 (0.04-0.60)	<0.001	0.35 (0.05-0.50)	<0.001
Lung left	0.46 (0.16-0.68)	<0.001	0.39 (0.13-0.63)	<0.001	0.41 (0.13-0.58)	<0.001
Breast right	0.33 (0.00-2.68)	0.008	0.33 (0.00-2.14)	0.008	0.33 (0.00-2.4)	0.008
Breast left	0.44 (0.01-0.84)	<0.001	0.42 (0.01-0.81)	<0.001	0.43 (0.01-0.83)	<0.001
Esophagus	0.72 (0.01-2.77)	0.002	0.70 (0.01-1.63)	<0.001	0.76 (0.01-2.24)	0.001

Clinical and oncologic outcomes have been reported in detail elsewhere (12). At the time of this analysis, median follow-up was 49.5 months (range, 34.7–68.8 months), and 5-year OS and 5-year PFS were 100 and 91.3%, respectively. No SM have been documented during follow-up.

## Discussion

As oncologic outcomes for mediastinal lymphoma have improved over time, there has been a renewed focus on treatment-related side effects. This is all the more important in a patient population who are typically diagnosed at a younger median age and have a more extended cancer natural history. Multiple dosimetric studies have provided evidence that PT offers a superior dose distribution in patients with mediastinal lymphoma relative to X-ray irradiation (12, 30, 31), which may lead to reduced acute and long-term toxicity. Of undisputed importance is the induction of SM, particularly lung and breast cancer. Majority of the applications are retrospective in nature and prospective trials are pending and oftentimes not feasible due to ethical complexities. Furthermore, for patients that have already been treated with PT, long-term data are still lacking, given the very long-term follow-up periods acquired to identify chronic toxicity including cardiovascular diseases or SM induction.

The bulk of clinical data comes from the X-ray era and partially from the 2D RT era and are therefore of limited applicability to modern RT techniques such as IMRT. One Dutch retrospective cohort study enrolled 3,905 HL patients treated with RT (primarily large-field irradiation techniques) and who had survived HL. In this cohort, 1,055 SM were diagnosed, resulting in a standardized incidence ratio of 4.6 compared to the general population, with the cumulative incidence of SM being 48.5% at 40 years after treatment vs. 19% in the general population. In this series, breast and lung cancer contributed the bulk of overall absolute excess risk increase (each 20%) (32). Furthermore, Moskowitz reported that the cumulative incidence of breast cancer by the age of 50 is comparable with the risk of BRCA1 mutation carriers for childhood HL survivors (33). Although data have to be interpreted with caution when extrapolating older studies using less advanced radiation techniques with current RT technology, these clinical data emphasize the importance of dose reduction, especially in young patients where the risk is even higher (34).

Regarding PT, clinical data are even more limited; however, in a retrospective matched-pair analysis of 558 patients, SM occurred in 7.5% after X-ray irradiation vs. 5.2 % after PT (35). Although the median follow-up is short (6.7 years), the extrapolated incidence rate of SM after X-ray irradiation was 10.3 cancers per 1,000 person-years compared to 6.9 cancers per 1,000 person-years following PT. Moreover, the interpretation of these results is also complicated by the heterogeneity of tumor and histologies, variations in combined modality approach, heterogeneity of radiation dose, and fractionation schemes used which may bias the results.

In an effort to evaluate the risk for SM induction following RT with modern techniques, we performed a pairwise comparison of the estimated individual risks for radiation-induced SMs after PT vs. X-ray irradiation for relevant organs in patients with mediastinal lymphoma using two different, well-established mechanistic calculation models. We showed that the calculated risks were significantly lower after PT compared to X-ray irradiation for all OARs investigated in this study (i.e., lungs, esophagus, and breast). Of note is that the risks in the aforementioned publications (32–34) may be higher, owing to the older radiation techniques, younger patient age, and consideration of the cumulative risk for all secondary malignancies, compared to an organ-, sex-, and age-specific risk estimation like our analysis. Several publications already confirmed a strong dependency of developing cancer at the age of exposure, including Hancock et al. who reported over three times of elevated risk for breast cancer when a patient below the age of 20 years was compared to older patients aged 20–29 years (36).

A retrospective comparative analysis of HL patients demonstrated that PT decreased the avoidable cancer incidence compared to X-rays by a factor of about 2 (3), using the IRCP-60 method. Similarly, our results show comparable values for risk reduction when using protons of 2.75–3.0 for lung cancer, 2.75–2.33 for breast cancer, and 1.25 for esophageal cancer (total risk according to Dasu), confirming previously reported results with a larger patient cohort. Another valuable metric, investigated by Rechner et al. (37), is the calculation of life years lost (LYL) attributable to the late effects after RT. This publication evaluated the risk for 22 patients and found that the use of PT significantly reduced LYL compared to IMRT. The primary drivers for LYL were heart failure, myocardial infarction, valvular heart disease, and breast and lung cancer, which again emphasize the importance of dose reduction to these OARs.

In two patients (nos. 2 and 20), the risk ratio (RR) according to the Schneider model for breast cancer on the right side was >1 and therefore higher with PT. Of note is that both patients were diagnosed with more right-lateralized mediastinal involvement and beam application was weighted more from this side, resulting in a lower dose to the left side (see Figure 2) and especially a lower dose to the heart. In both patients, for example, this was considered more important since these patients had already suffered from grade 2 chronic heart failure after chemotherapy. In general, this demonstrates that relative risks are associated not only with treatment planning and technique factors but also with patient-specific geometry and tumor location. Nevertheless, these two patients were treated with PT due to the significant improvement in other thoracic OARs. Finally, the two patients (nos. 10 and 18) with a higher RR for esophageal cancer induction were both patients with cervical and upper mediastinal involvement, where the dose to the esophagus was higher with PT, owing to the beam arrangements. Nevertheless, PT was chosen in these patients due to better sparing of other OARs (breast and heart), where risk for SM or long-term toxicity is more relevant.

Overall, most organs at risk demonstrated significant dosimetric improvements across the cohort analyzed. However, tumor location and patient geometry, on rare occasions, led to improvements in dose to certain organs. As a result, clinician judgment must be used on a case-by-case basis when deciding between radiation modalities that may have variable improvements between OAR doses, that is, if a given proton plan yields reduction in heart and lung dose but higher breast dose relative to a comparative IMRT plan, clinical factors will need to be weighed by the radiation oncologist to choose the plan most likely to optimize patient clinical outcome. Notably, there are several limitations for modeling radiation-induced carcinogenesis: Firstly, both models applied in this analysis use data derived from epidemiological studies which *per se* have uncertainties: factors like whole-body exposure in atomic bomb survivors vs. local dose exposure in radiotherapy might reduce comparability (38). Moreover, RBE may vary in PT, and this effect is currently not considered in these models but is also not taken into consideration in standard clinical PT (use of constant RBE of 1.1).

Nevertheless, the strength of the two models is the inclusion of factors for cell killing as well as repair and repopulation, which reflect the non-linear dose–response relationship that is well known for SM induction (39).

Apart from all these factors, real patient data (that need decades to be collected) will also suffer from variables that influence certainty, e.g., variation of target size and tumor location between patients, as well as the use of different planning/optimization techniques and constraints. In this context, using risk ratios in a pairwise comparison of different modalities may be very useful when ranking RT modalities like proton and photon irradiation in a given patient cohort.

As proposed by a current guideline of the ILROG (40), PT is an attractive treatment option which should be discussed for lymphoma patients, especially if mediastinal involvement is present. Nevertheless, the potential benefit is variable and dependent on many factors including age, gender, tumor location, and patient-specific comorbidities. This specific radiation modality should be discussed on a “case-by-case” basis and, if found to be warranted, patients should be treated at PT facilities with sufficient expertise (41). At our facility, all lymphoma patients treated with PT are placed on a prospective registry study with long follow-up to investigate long-term toxicities like cardiac events or SM.

Furthermore, the American Cancer Society, the American College of Radiology, and the Society of Breast Imaging recommend annual screening by breast magnetic resonance imaging as an intensified screening for breast cancer, especially for patients treated at an age <30 years, similar to the already established screening for high-risk patients with a BRCA1 mutation (42, 43).

## Conclusion

Proton therapy for patients diagnosed with mediastinal lymphoma offers a dramatic dose reduction to surrounding thoracic OARs. Based on the multiple radiobiological models utilized in the present study, PT is estimated to reduce SM risk for lung and breast tissue. Future research will include a long-term follow-up of patients treated at experienced facilities to identify the “real” risk of secondary malignancies in this patient population.

## Data Availability Statement

The datasets generated for this study are available on request to the corresponding author.

## Ethics Statement

The studies involving human participants were reviewed and approved by Ethikkommission Universität Heidelberg. Written informed consent for participation was not required for this study in accordance with the national legislation and the institutional requirements.

## Author Contributions

LK, PHa, CL, and MS performed the data collection. LK, JL, and JH-R were responsible for writing of the original draft preparation. LK, BN, FW, PHo, JD, and KH performed patient treatment and clinical assessments and were responsible for radiooncological follow-up documentation. LK, PHa, and JH-R performed the statistical analysis. LK and JH-R conceived the study and participated in its design and coordination. All the authors were responsible for data interpretation, participated in the manuscript revisions, and approved the final manuscript.

## Conflict of Interest

The authors declare that the research was conducted in the absence of any commercial or financial relationships that could be construed as a potential conflict of interest.
